# The application of custom-made 3D-printed titanium augments designed through surgical simulation for severe bone defects in complex revision total hip arthroplasty

**DOI:** 10.1186/s10195-022-00656-5

**Published:** 2022-08-06

**Authors:** Yanchao Zhang, Zhisen Gao, Bohan Zhang, Yinqiao Du, Haiyang Ma, Yuyu Tang, Yang Liu, Yonggang Zhou

**Affiliations:** 1 Department of Orthopedics, the First Medical Center, Chinese PLA General Hospital, Beijing, 100853 China; 2 Medical School of Chinese PLA, 100048 Beijing, China; 3 Senior Department of Orthopedics, The Fourth Medical Center of PLA General Hospital, Beijing, 100048 China; 4 Medical School of Nankai University, Tianjin, 300071 China

**Keywords:** Total hip arthroplasty, Revision, Three-dimensional printed augment, Bone defect

## Abstract

**Background:**

With the development of radiology and three-dimensional (3D) printing technology, custom-made 3D-printed titanium augments have been more widely used. However, the radiological and clinical outcomes of custom-made augments lack reports. To better understand the clinical effect of using 3D-printed titanium augments and the significance of accurate placement, the aim of this study was to assess the outcomes when using custom-made 3D-printed titanium augments and to validate the idea that surgical simulation should be done before designing custom-made augments.

**Methods:**

A retrospective review was conducted on 31 surgical simulations and revision total hip arthroplasties using custom-made 3D-printed titanium augments. The safe zone, cup position, and hip rotation center were measured on anteroposterior radiographs. Clinical outcomes were assessed with a mean 21.1 months of follow-up.

**Results:**

All patients were positioned within the safe zone, and none of the acetabular cups nor the custom-made augments had any evidence of migration at the latest follow-up. A strong correlation was found between the planned cup position and the postoperative position. The average vertical position of the center of rotation was significantly increased from 3.55 cm to 2.35 cm. The mean Harris Hip Score was increased from 40.81 preoperatively to 65.46 postoperatively. Complications included gait abnormality, groin pain, fracture of the greater trochanter, and partial palsy of the sciatic nerve. However, patient satisfaction reached 92.3%.

**Conclusion:**

Surgical simulations help to design custom-made augments accurately and improve surgical plans. Acetabular components supported with custom-made 3D-printed augments is a useful method to bridge severe bone deficiencies. In this study, both the radiologic results and clinical outcomes were favorable.

**Level of evidence:**

Level 4.

**Supplementary Information:**

The online version contains supplementary material available at 10.1186/s10195-022-00656-5.

## Introduction

Revision total hip arthroplasties (rTHAs) in the presence of severe acetabular defects can be a big challenge. In this circumstance, the use of a large cementless hemispheric cup is the first choice for most investigators, and has become even more versatile with the introduction of the extra-large hemispheric cup [1–7]. When a hemispheric cup could not be used because of instability or a lack of sufficient cup coverage, porous metal augments were applied to acetabular reconstructions. Both mid-term follow-up and long-term follow-up of porous metal augments have shown excellent survivability [8–10].

Recently, with the development of radiology and three-dimensional (3D) printing technology, custom-made 3D-printed titanium augments have been more widely used. The custom-made augment inherits the advantages of the porous metal augment and is more accurate than it. The strengths of custom-made augments include avoiding excessive reaming of the acetabulum due to a poor match, which can preserve the bone stock and simplify the surgery; matching the bone defects perfectly and providing the acetabular component with sufficient initial stability; and their ability to be printed according to the anatomical features of the patients. Thus, they also serve to reconstruct the hip rotation center and restore the hip biomechanics after THAs. Additionally, 3D-printed augments have personalized screw trajectories. Full-length insertion screws can be used, helping to avoid neurovascular bundle damage. Theoretically, custom-made 3D-printed titanium augments should have a more promising outcome than porous metal augments.

However, in the study of Baauw et al. [11], they found that 43.8% of custom-made implants were malpositioned owing to inaccurate planning (a lack of surgical simulation of the 3D-printed hip model). The objectives of our study were (1) to validate the idea that surgical simulation of the 3D-printed hip model should be done before designing custom-made augments and (2) to assess the radiological and clinical outcomes of using custom-made 3D-printed titanium augments.

## Materials and methods

### Patients and methods

After institutional review board approval, we retrospectively reviewed our revision THAs from August 2017 to November 2021. Patients were included if they received custom-made 3D-printed titanium augments (pore size 600 to 800 μm and porosity 60% to 80%), but they were excluded if they had prefabricated augments besides custom-made augments. Finally, 30 patients (31 hips) were included in our cohort. None of the patients were lost to follow-up.

Demographic data were collected on all patients, as described in Table 1. There were 10 men and 20 women in this study. The mean age at the time of rTHA was 53.7 (33 to 79) years. The mean body mass index (BMI) was 25.2 (17.1 to 38.9) kg/m^2^. Among the cohort, four patients were followed up for less than 3 months. Due to their short recovery times, they were still taking NSAIDs, and two of them still could not get off their crutches. Those four patients were not included in our clinical outcome assessment, and the mean follow-up was 21.7 months (4.3 to 51.2).

**Table 1:** Demographic characteristics

Case number	Gender	Age (years)	BMI	Primary diagnosis	Reason for revision	Surgical side	Paprosky classification	Follow-up (months)
1	F	66	28.76	AVN	AL	R	3B	53.27
2	M	56	22.31	AS	PJI	L	2C	51.80
3	F	43	23.92	AVN	PW; AL	L	2B	41.77
4	F	42	24.03	AF	AL	R	2A	41.30
5	F	46	27.34	AVN	AL	L	2B	39.67
					AL	R	2B	39.67
6	F	38	18.82	GCTB	AL	R	3A	38.97
7	M	63	24.96	AVN	AL	L	3A	32.03
8	F	70	26.37	OA	AL	L	3A	31.80
9	M	76	26.57	AVN	PW; AL	L	3B	29.40
10	F	52	25.07	AVN	PJI	L	2B	29.23
11	F	33	17.09	DDH	PW	L	1	26.60
12	M	67	22.49	FNF	PJI	R	2C	16.93
13	M	40	38.87	AF	AL	R	2B	15.57
14	F	77	22.19	AF	PJI	R	3B	15.40
15	F	63	25.91	FNF	AL	L	3B	13.77
16	M	79	25.71	AVN	PW; AL	L	3A	8.87
17	M	52	30.49	AVN	AL	R	3B	8.23
18	F	67	24.44	AVN	CD	L	3B	7.70
19	F	34	34.01	AF	AL	R	2A	6.60
20	F	41	21.37	OA	AL	L	3A	6.23
21	F	47	26.81	AF	PW; AL	L	2A	6.13
22	M	65	30.37	AVN	PW	L	1	6.07
23	F	53	26.57	AVN	PW; AL	R	2B	5.20
24	M	46	18.11	OA	PW; AL	L	3B	4.97
25	F	43	21.91	AVN	PW; AL	R	2B	4.50
26	F	64	26.56	OA	PW; AL	L	2A	4.27
27	F	44	19.31	RA	AL	L	3B	2.73
28	F	44	23.44	AVN	PJI	L	2C	2.63
29	F	52	23.44	DDH	AL	R	3B	1.33
30	M	48	28.69	AVN	PW; AL	L	2B	0.77

*M* male,* F* female,* AVN* avascular necrosis of femoral head,* AF* acetabular fracture,* FNF* femoral neck fracture,* OA* osteoarthritis,* AS* ankylosing spondylitis,* GCTB* giant-cell tumor of bone,* DDH* developmental dysplasia of the hip,* RA* rheumatoid arthritis,* PJI* periprosthetic joint infection,* PW* polyethylene wear,* AL* aseptic loosening,* CD* central dislocation,* L* left,* R* right

### Indications for building 3D-printed hip models

Acetabular bone deficiencies were categorized according to the method of Paprosky et al. [12] using pre-revision anteroposterior (AP) radiographs. When the lead surgeon could not directly judge the bony condition from radiographs, computed tomography (Brilliance 256-slice iCT; Philips Healthcare, Cleveland, OH, USA) was needed to provide three-dimensional structural information. Based on the CT data, a 3D-printed hip model (EOS M280 3D printer, EOS, Krailling, Germany) was established, and a simulated surgery was then performed on it. After this procedure, prosthesis selection, prosthesis sizing, the number of augments, and the positions of augments were determined. This procedure is described in the next section.

### Surgical simulation

**Fig. 1:**
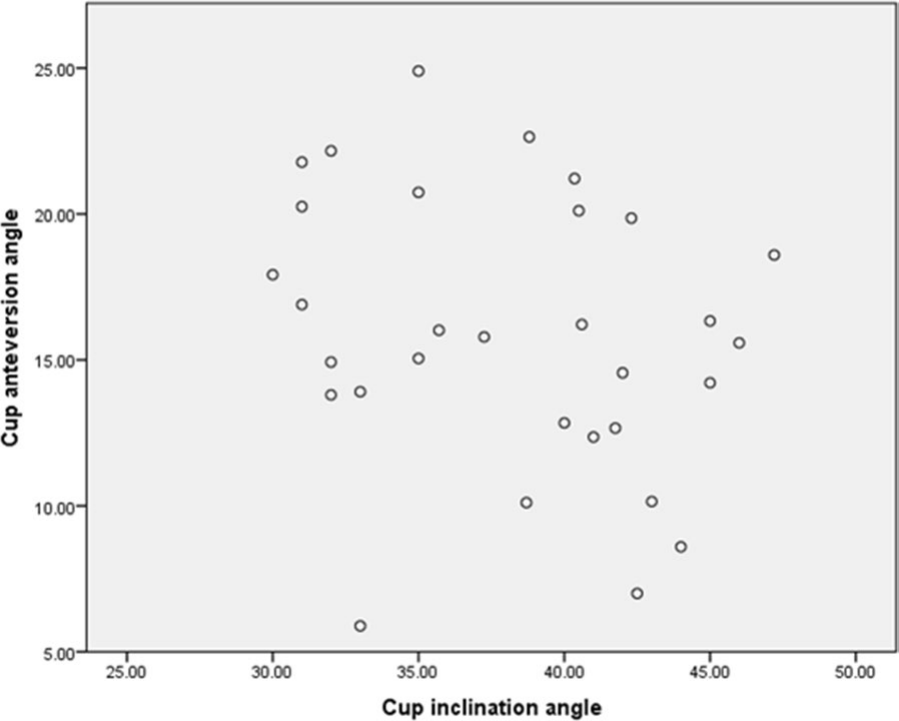
Parametric measures. **a*** S* short axis of the ellipse;* Lo* long axis of the ellipse;* white arrow* inclination angle; anteversion < arcsin(S/Lo). **b** Measuring the position of the hip rotation center.* HCOR* horizontal position of the center of rotation;* VCOR* vertical position of the center of rotation. **c** Measuring the limb length discrepancy

Surgical simulation was performed by the surgical performer in each case. Standard instruments for THA were prepared. The surgeon reamed the acetabulum to the appropriate size in successive increments, replicating the intraoperative process. Post reaming, a trial cup was secured and impacted where possible to achieve sufficient contact area with the host bone and to acquire adequate component stability. In unstable cases, plasticine was shaped and inserted into the acetabular bone deficiencies. Finally, our engineer redesigned custom-made augments according to the shaped plasticine. During this procedure, we checked the match between the 3D-printed augments and pelvic model several times (Fig. 1: case 16).

### Surgical procedure

The 3D-printed pelvis model and custom-made 3D-printed titanium augments were sterilized and prepared for use in surgery. After preoperative preparation, the failed acetabular component was removed through a posterolateral hip approach. The lead surgeon debrided the remaining acetabulum and reassessed acetabular bone defects by comparison with the 3D-printed hip model. Then the custom-made augments were inserted into the patient and fixed with screws as planned. After that, the acetabulum was reamed with the planned degrees of inclination and anteversion in successive increments from a smaller size to a diameter that was 1 mm or 2 mm less than the planned acetabular cup size. Post reaming, the grinding debris was removed and a trial cup was tested. If the cup position, stability, and contact area with the host bone were thought to be acceptable, bone cement was applied to the surface between the cup and augment and then a porous-coated cementless acetabular shell was secured and impacted with adequate press-fit and screws. Finally, the stability of the cup was evaluated. Table 2 describes the surgical details.

**Table 2:** Surgical details

Case number	Cup size (mm)	Number of augments	Position of augments	ASA score	Femoral stem revision	Surgical period (h)	Blood loss (ml)
1	72	1	S	2	No	4.0	1200
2	58	2	A; P	2	Yes	5.5	3000
3	52	1	S	1	No	2.8	600
4	58	1	P	1	No	2.5	300
5 (L)	58	1	PI	2	No	2.6	1400
5 (R)	60	1	AS	2	No	2.6	800
6	62	1	PS	1	No	4.0	1500
7	62	1	PS	2	No	3.0	600
8	66	1	S	2	Yes	3.2	800
9	68	2	S; S	2	Yes	3.0	600
10	58	1	S	2	No	3.0	500
11	50	1	S	1	No	2.4	1000
12	64	1	M	1	Yes	4.5	1000
13	64	1	P	1	Yes	3.5	800
14	72	1	S	2	Yes	4.4	1200
15	58	1	S	2	Yes	2.6	800
16	66	3	PS; PI; AI	2	No	5	1000
17	60	1	S	1	Yes	4.6	1500
18	66	1	S	2	No	3.5	800
19	56	1	PS	1	Yes	2.5	600
20	56	1	PS	2	Yes	2.5	500
21	54	1	PS	1	Yes	2.6	800
22	64	2	S; PI	2	No	2.6	600
23	54	1	S	2	Yes	3.5	600
24	72	2	S; AS	1	Yes	3.2	1500
25	54	2	S; PI	1	No	3.3	800
26	64	2	S; PS	2	Yes	3.7	800
27	58	2	S; S	2	No	3.4	800
28	66	2	S; PI	3	Yes	3.6	1000
29	58	1	PS	2	No	4.0	800
30	60	3	S; PI; AI	2	Yes	3.0	800

*L* left,* R* right,* S* superior,* A* anterior,* P *posterior,* PI* posteroinferior,* AS* anterosuperior,* PS* posterosuperior,* M* medial,* I* inferior,* AI* anteroinferior,* ASA* American Society of Anesthesiologists

### Rehabilitation after operation

On the first day after surgery, patients were encouraged to walk with crutches and partial weight-bearing was allowed. A full weight-bearing gait was permitted at 6 weeks postoperatively. In the meantime, short-term antibiotic prophylaxis with a third-generation cephalosporin and low-molecular-weight heparin were applied to all patients.

### Radiological assessment

**Fig. 2:**
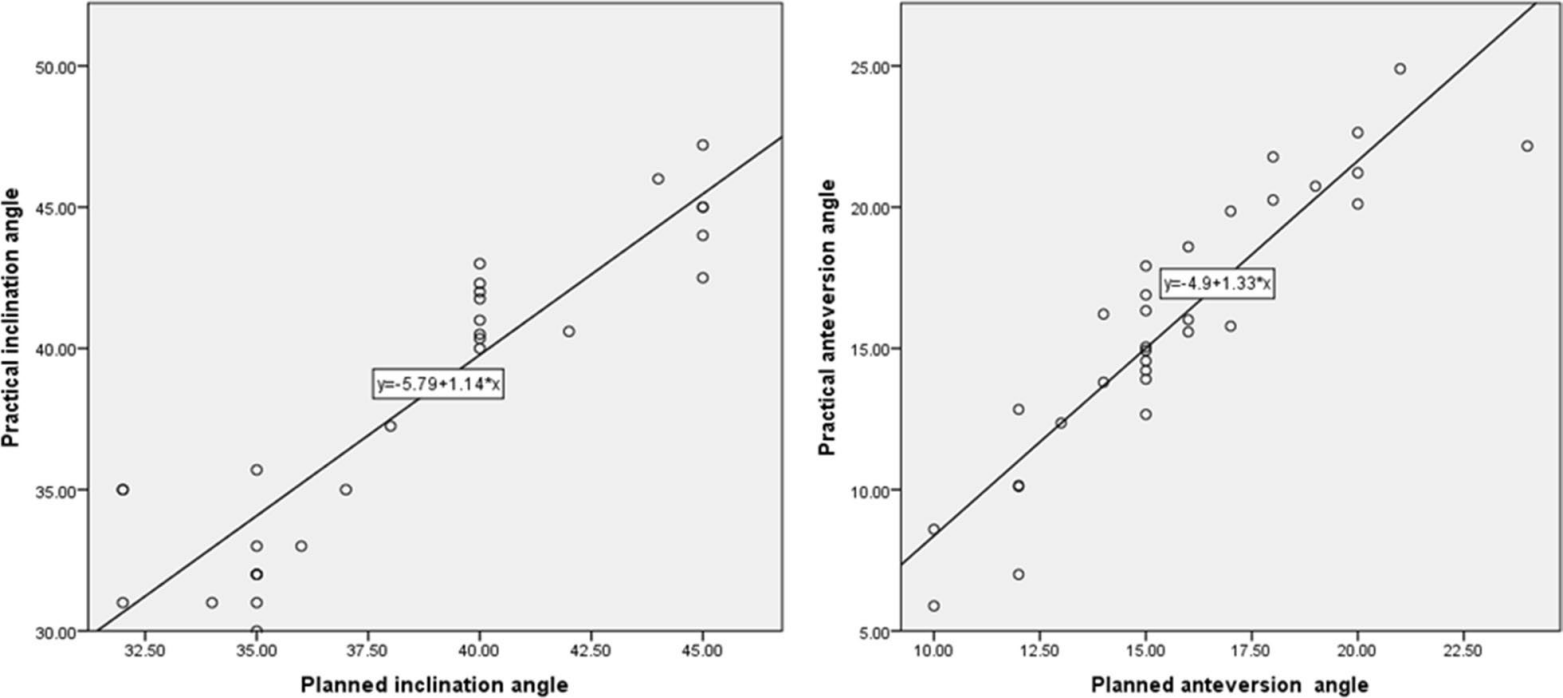
Scatter plot of each hip’s inclination and anteversion angles

Standard AP radiographs and lateral radiographs were done before the revision THA, at 3 postoperative days, and routinely at every visit. The safe zone [13] and the DeLee and Charnley classification [14] were used to assess the cup fixation, based on the AP radiographs. Radiolucencies were assessed at the cup–bone interface and the augment–bone interface. The osseointegration of acetabular shells and augments was evaluated using the Moore criteria [15] and the Abolghasemian criteria [16], respectively. More than 3 mm of vertical or horizontal migration, a change in inclination angle of over 5°, or less than two signs of osseointegration indicated radiographic failure. The hip rotation center was measured on the preoperative and postoperative AP radiographs based on the modified method of Ranawat et al. [17]. The leg length discrepancy (LLD) was described as the distance from the base of the teardrop to the corresponding tip of the lesser trochanter. When it was violated due to medialization of the acetabular component, the teardrop was identified by reference to the preoperative radiograph. Figure 2 illustrates the measurement process. All image data were assessed by two experienced observers independently.

### Statistical analysis

Interobserver and intraobserver reliability were assessed by intraclass correlation coefficients (ICCs) for the measurements. Two observers who did not participate in the operations and were blind to the clinical results measured all the parameters. For the intraobserver reliability, the interval between measurements was at least 1 month. Both the intraobserver reliabilities were more than 0.9. The interobserver variance also had an ICC of more than 0.8, which indicated high agreement between all the measurements(see Additional file 1). The mean values of the two observers were used for analysis.

The mean values and ranges were calculated for demographic data and are presented as the mean ± standard deviation with the range. Categorical variable values are presented as percentages. The paired* t*-test was used to  determine the statistical significance of the preoperative vs. postoperative difference in the hip rotation center. Statistical significance was defined as *P* < 0.05. All statistical analyses were conducted with SPSS version 24.0 (IBM Inc., Armonk, New York).

## Results

### Radiologic results

**Fig. 3:**
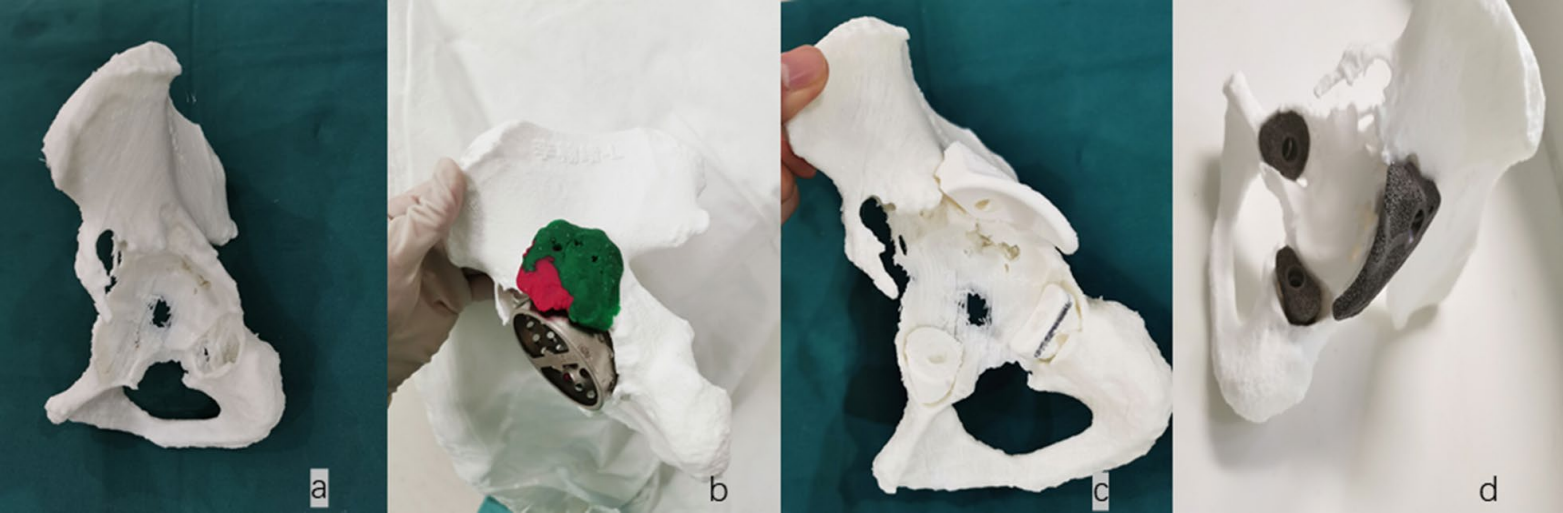
The planned and the practical values of cup inclination and anteversion

Measurements using the AP radiographs taken on postoperative day 3 indicated that all the patients were positioned within the safe zone (Fig. 3). The mean values were 38.12° for inclination and 15.91° for anteversion. One patient showed a local radiolucency in zone I according to the DeLee and Charnley classification system. However, this was found to be a tiny contained bone deficiency in the  roof of the acetabulum and the cup was tested and found to be stable in surgery. At his 1-year follow-up, the radiolucency blurred. In the remaining patients, no radiolucent lines were observed at either the cup–bone or the augment–bone interface during their follow-up. None of the acetabular cups nor 3D-printed augments had any evidence of migration in our study.

**Fig. 4:**
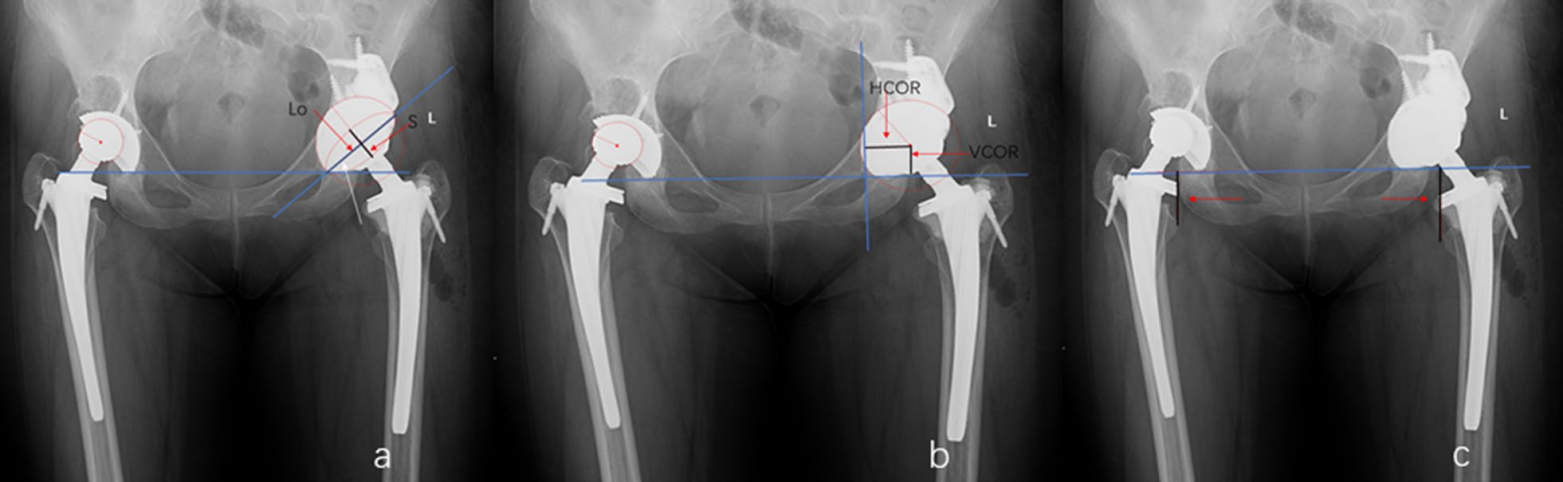
Surgical simulation. **a** 3D-printed hip model. **b** Acetabular bone deficiency in the hip model filled with plasticine. **c** 3D-printed plastic augments. **d** 3D-printed porous-metal augments

The correlation between the planned and the postoperative cup orientations is shown in Table 3. The planned cup inclination and anteversion values did not show a significant difference from their postoperative values, and strong correlations were found between the planned and postoperative values (Fig. 4).

**Table 3:** Planned and practical inclination and anteversion

	Planned	Practical	*P* value^a^	*R* value
Inclination	38.55 ± 4.15°	38.12 ± 5.20°	0.294	0.909
Anteversion	15.68 ± 3.24°	15.91 ± 4.70°	0.565	0.914

^a^Planned vs. practical

Table 4 lists the positions of the center of rotation (COR). For the vertical position of the COR (VCOR), there was a difference between the surgical side and the contralateral side preoperatively (3.71 ± 1.66 cm vs. 1.78 ± 1.17 cm). A difference was also found when comparing the preoperative VCOR with the postoperative VCOR. The mean value was significantly improved from 3.71 ± 1.66 cm to 2.33 ± 1.04 cm. No such differences were found for the horizontal position of the COR (HCOR). Although there was still a deviation in the VCOR between the surgical side and the contralateral side postoperatively, the position of the COR was corrected (it was closer to the position of the COR on the contralateral side).

**Table 4:** Position of COR

	Preop values (cm)			Postop values (cm)			*P*^a^
	Surgical side	Contralateral side	*P*	Surgical side	Contralateral side	*P*	
VCOR	3.71 ± 1.66	1.78 ± 1.17	< 0.001*	2.33 ± 1.04	1.78 ± 1.17	0.032*	< 0.001*
HCOR	3. 43 ± 1.19	3.59 ± 0.55	0.689	3.48 ± 0.93	3.59 ± 0.55	0.799	0.799

*Preop* preoperative;* Postop* postoperative

^*^P < 0.05

^a^Preoperative surgical side vs. postoperative surgical side

### Clinical outcomes

As outlined in Table 5, the mean Harris Hip Score (HHS) was improved from 39.98 preoperatively to 69.22 at the final follow-up (*P* < 0.001). The mean LLD was 0.337 cm, but in one case the LLD exceeded 1 cm. One patient presented with sciatica when the operative hip was flexed beyond 90°, but this symptom was gradually improving. One patient had a fracture of the greater trochanter during surgery. Three patients had slight groin pain occasionally. However, no infections, dislocations, aseptic loosening, or acetabular periprosthetic fractures were found in our study. In the gait assessment at the latest follow-up, 23 patients had a limp, of which 14 were classified as slight, 3 as moderate, and 6 as severe. However, at the end of the follow-up, patient satisfaction reached 92.3%.

**Table 5:** Clinical results

Case number	Preop HHS	Postop HHS	LLD (cm)	Gait	Other complications	Satisfaction survey
1	53	59	− 0.026^a^	M		Sa
2	42	72	− 0.223	Sl		Sa
3	56	65	0.647	Se		Ds
4	46	88	0.434	No		Sa
5 (L)	55	69	− 0.277	Sl		Sa
5 (R)	57	70	0.277			
6	36	50	0.369	Se		Sa
7	58	90	0.256	No		Sa
8	48	56	0.118	Se	FGT	Ds
9	45	73	0.473	Sl	GP	Sa
10	37	71	0.709	Sl	GP	Sa
11	55	92	0.638	No		Sa
12	17	61	0.438	M		Sa
13	44	76	0.061	Sl		Sa
14	15	70	0.753	Sl		Sa
15	4	89	0.291	No		Sa
16	51	63	0.601	M	GP	Sa
17	41	52	1.217	Se	S	Sa
18	2	69	0.730	Sl		Sa
19	59	77	− 0.340	Sl		Sa
20	19	51	0.003	Se		Sa
21	40	70	0.361	Sl		Sa
22	30	75	0.031	Sl		Sa
23	60	74	0.632	Sl		Sa
24	5	48	0.207	Se		Sa
25	47	68	0.238	Sl		Sa
26	57	71	0.493	Sl		Sa

*L* left,* R* right,* Preop* preoperative,* Postop* postoperative,* HHS* Harris Hip Score,* Sl* slight,* M* moderate,* Se* severe,* FGT* fracture of the greater trochanter,* GP* groin pain,* S* sciatica,* Sa* satisfactory,* Ds* dissatisfactory

^a^A negative LLD means that the surgical side was shorter than the contralateral side

## Discussion

**Fig. 5:**
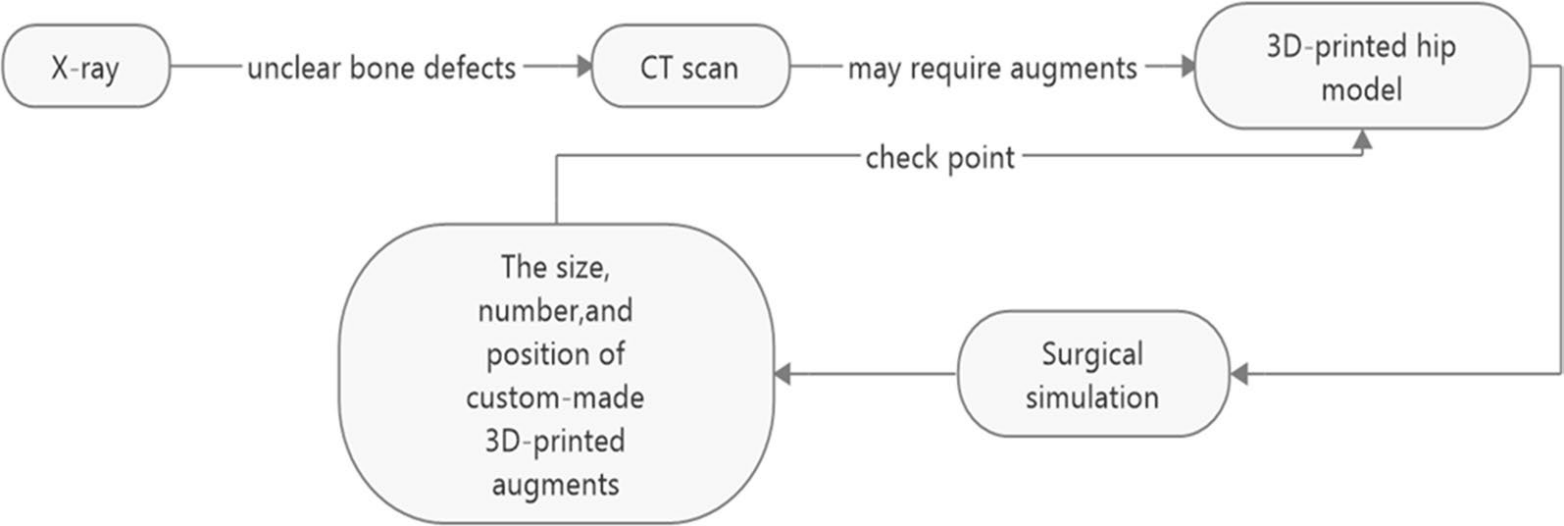
The workflow of designing custom-made 3D-printed titanium augments

In our study, we found that surgical simulation provided support for the approaches and techniques required. Figure 5 shows the workflow of designing custom-made 3D-printed titanium augments. Using life-size 3D-printed models allows a clear understanding of acetabular bone deficiency. In an article by Jiang et al. [18], a pilot study using patient-specific 3D-printed models as a method of streamlining the preoperative planning process was presented. Kavalerskiy et al. [19] concluded that the number and type of planned augments were the same as the number and type of planned used augments in all 14 cases. In our study, we found the final cup position to have a strong correlation with the plan from surgical simulation. Similar to the result of Li et al. [20], the acceptable deviation might derive from the insufficient proximal exposure or the change in patient position during surgery.

The implant positions play a major role in THA instability [21]. In 1978, Lewinnek et al. [13] defined their “safe zone” as 5–25° anteversion and 30–50° inclination, based on AP radiographs. However, this has remained controversial. In the study of Abdel et al. [22], nearly 60% of the unstable THA cases had satisfactory cup positions. Esposito et al. [23] and Reize et al. [24] reported similar results. Even Dorr et al. [25] declared that the safe zone was dead. Lewinnek’s safe zone is not perfect, but it is a simple and science-based method. Based on Lewinnek’s idea, Reina et al. [26] put forward the concept of a “target zone,” which was a narrowed safe zone that showed good ability to distinguish unstable cases from stable cases. However, CT radiographs are needed to use the target zone. In our hospital, postoperative CT scans are not routine examinations during patient follow-up.

The restoration of the COR to an anatomic position is related to satisfactory functional outcomes [27–31]. In our study, the VCOR decreased on average by 1.50 cm compared with its preoperative value on the surgical side. This indicates that the 3D-printed augment is a useful method to restore the anatomical COR.

Among the cohort, six patients had a severe gait abnormality. Gait abnormality is influenced by many factors, including leg length discrepancy, poor gluteus medius strength, and pain [32, 33]. Confounding factors are numerous and difficult to get rid of. After the patients with severe limping for special reasons, including the osteolysis of the greater trochanter, strength of gluteus medius, LLD, and knee stiffness were excluded, our study demonstrated that restoring anatomical COR helped patients to achieve a better gait than before surgery. However, more research is needed to verify the validity of this point.

## Limitation

There are several limitations of this study. First, being a retrospective study design, it is never as ideal as a randomized controlled trial for comparisons of this technique with other reconstruction techniques. Second, the small sample size and relatively short follow-up duration may have limited the interpretable power. However, this is one of the largest studies on 3D-printed augments in terms of sample size. Third, the femoral side also has an impact on postoperative gait, but in this study we paid more attention to the relationship between the position of the COR and lameness. Fourth, further follow-up of our patients is needed to demonstrate the long-term efficacy of our custom-made 3D-printed augments. Finally, there is no clear definition of “complex revision THA;” instead, each surgeon decides when to print a hip model and simulate a surgery based on his or her clinical experience and skill level.

## Conclusion

Surgical simulations aid the accurate design of custom-made augments and improve surgical plans. Acetabular components supported with custom-made 3D-printed augments are a good way to bridge severe bone deficiencies. In our study, most patients showed satisfactory results in both radiologic and clinical assessments, but further follow-ups are needed to demonstrate long-term results.

## Supplementary information

**Additional file 1. **Intraobserver and interobserver evaluations.

## Data Availability

The data that support the findings of this study are available from the First Medical Center of PLA General Hospital, but restrictions apply to the availability of these data, which were used under license for the current study and so are not publicly available. Data are, however, available from the authors upon reasonable request and with the permission of the First Medical Center of PLA General Hospital.

## Abbreviations

3D: Three-dimensional
THA: Total hip arthroplasty
HCOR: Horizontal position of the center of rotation
VCOR: Vertical position of the center of rotation
HHS: Harris Hip Score
LLD: Leg length discrepancy
