# Supplementary material for: The application of custom-made 3D-printed titanium augments designed through surgical simulation for severe bone defects in complex revision total hip arthroplasty
Source: J Orthop Traumatol. 2022 Aug 6;23:37. doi: 10.1186/s10195-022-00656-5 (PMC9357241; doi:10.1186/s10195-022-00656-5)
Supplement: Supplementary file 1 — Additional file 1. Intraobserver and interobserver evaluations. [file 10195_2022_656_MOESM1_ESM.doc]

1.	Postoperative cup inclination
Consistency of observer 1
RELIABILITY
  /VARIABLES=observer1first observer1second
  /SCALE('ALL VARIABLES') ALL
  /MODEL=ALPHA
  /ICC=MODEL(RANDOM) TYPE(CONSISTENCY) CIN=95 TESTVAL=0.

Reliability
Scale: ALL VARIABLES
Case Processing Summary	
	N	%	
Cases	Valid	31	100.0	
	Excludeda	0	.0	
	Total	31	100.0	

a. Listwise deletion based on all variables in the procedure.	

Reliability Statistics	
Cronbach's Alpha	N of Items	
.992	2	


Intraclass Correlation Coefficient	
	Intraclass Correlationb	95% Confidence Interval	F Test with True Value 0			
		Lower Bound	Upper Bound	Value	df1			
Single Measures	.983a	.966	.992	119.573	30			
Average Measures	.992	.983	.996	119.573	30			

Consistency of observer 2
RELIABILITY
  /VARIABLES=observer2first observer2second
  /SCALE('ALL VARIABLES') ALL
  /MODEL=ALPHA
  /ICC=MODEL(RANDOM) TYPE(CONSISTENCY) CIN=95 TESTVAL=0.

Reliability
Scale: ALL VARIABLES


Case Processing Summary	
	N	%	
Cases	Valid	31	100.0	
	Excludeda	0	.0	
	Total	31	100.0	

a. Listwise deletion based on all variables in the procedure.	

Reliability Statistics	
Cronbach's Alpha	N of Items	
.995	2	


Intraclass Correlation Coefficient	
	Intraclass Correlationb	95% Confidence Interval	F Test with True Value 0			
		Lower Bound	Upper Bound	Value	df1			
Single Measures	.991a	.981	.996	214.865	30			
Average Measures	.995	.990	.998	214.865	30			

Consistency between observers
RELIABILITY
  /VARIABLES=meanvalueofobserver1 meanvalueofobserver2
  /SCALE('ALL VARIABLES') ALL
  /MODEL=ALPHA
  /ICC=MODEL(RANDOM) TYPE(CONSISTENCY) CIN=95 TESTVAL=0.

Reliability
Scale: ALL VARIABLES

Case Processing Summary	
	N	%	
Cases	Valid	31	100.0	
	Excludeda	0	.0	
	Total	31	100.0	

a. Listwise deletion based on all variables in the procedure.	

Reliability Statistics	
Cronbach's Alpha	N of Items	
.993	2	

Intraclass Correlation Coefficient	
	Intraclass Correlationb	95% Confidence Interval	F Test with True Value 0			
		Lower Bound	Upper Bound	Value	df1			
Single Measures	.985a	.969	.993	133.836	30			
Average Measures	.993	.985	.996	133.836	30			

2. Postoperative cup anteversion
Consistency of observer 1
RELIABILITY
  /VARIABLES=observer1first observer1second
  /SCALE('ALL VARIABLES') ALL
  /MODEL=ALPHA
  /ICC=MODEL(RANDOM) TYPE(CONSISTENCY) CIN=95 TESTVAL=0.

Reliability

Scale: ALL VARIABLES

Case Processing Summary	
	N	%	
Cases	Valid	31	100.0	
	Excludeda	0	.0	
	Total	31	100.0	

a. Listwise deletion based on all variables in the procedure.	

Reliability Statistics	
Cronbach's Alpha	N of Items	
.992	2	


Intraclass Correlation Coefficient	
	Intraclass Correlationb	95% Confidence Interval	F Test with True Value 0			
		Lower Bound	Upper Bound	Value	df1			
Single Measures	.985a	.969	.993	133.097	30			
Average Measures	.992	.984	.996	133.097	30			

Consistency of observer 2
RELIABILITY
  /VARIABLES=observer2first observer2second
  /SCALE('ALL VARIABLES') ALL
  /MODEL=ALPHA
  /ICC=MODEL(RANDOM) TYPE(CONSISTENCY) CIN=95 TESTVAL=0.

Reliability

Scale: ALL VARIABLES

Case Processing Summary	
	N	%	
Cases	Valid	31	100.0	
	Excludeda	0	.0	
	Total	31	100.0	

a. Listwise deletion based on all variables in the procedure.	

Reliability Statistics	
Cronbach's Alpha	N of Items	
.991	2	


Intraclass Correlation Coefficient	
	Intraclass Correlationb	95% Confidence Interval	F Test with True Value 0			
		Lower Bound	Upper Bound	Value	df1			
Single Measures	.982a	.964	.992	113.174	30			
Average Measures	.991	.982	.996	113.174	30			

Consistency between observers
RELIABILITY
  /VARIABLES=meanvalueofobserver1 meanvalueofobserver2
  /SCALE('ALL VARIABLES') ALL
  /MODEL=ALPHA
  /ICC=MODEL(RANDOM) TYPE(CONSISTENCY) CIN=95 TESTVAL=0.

Reliability

Scale: ALL VARIABLES

Case Processing Summary	
	N	%	
Cases	Valid	31	100.0	
	Excludeda	0	.0	
	Total	31	100.0	

a. Listwise deletion based on all variables in the procedure.	

Reliability Statistics	
Cronbach's Alpha	N of Items	
.994	2	


Intraclass Correlation Coefficient	
	Intraclass Correlationb	95% Confidence Interval	F Test with True Value 0			
		Lower Bound	Upper Bound	Value	df1			
Single Measures	.988a	.974	.994	159.655	30			
Average Measures	.994	.987	.997	159.655	30			

3.VCOR of preoperative surgical side
Consistency of observer 1
RELIABILITY
  /VARIABLES=observer1first observer1second
  /SCALE('ALL VARIABLES') ALL
  /MODEL=ALPHA
  /ICC=MODEL(RANDOM) TYPE(CONSISTENCY) CIN=95 TESTVAL=0.

Reliability

Scale: ALL VARIABLES

Case Processing Summary	
	N	%	
Cases	Valid	31	100.0	
	Excludeda	0	.0	
	Total	31	100.0	

a. Listwise deletion based on all variables in the procedure.	


Reliability Statistics	
Cronbach's Alpha	N of Items	
1.000	2	


Intraclass Correlation Coefficient	
	Intraclass Correlationb	95% Confidence Interval	F Test with True Value 0			
		Lower Bound	Upper Bound	Value	df1			
Single Measures	.999a	.998	1.000	2228.416	30			
Average Measures	1.000	.999	1.000	2228.416	30			

Consistency of observer 2
RELIABILITY
  /VARIABLES=observer2first observer2second
  /SCALE('ALL VARIABLES') ALL
  /MODEL=ALPHA
  /ICC=MODEL(RANDOM) TYPE(CONSISTENCY) CIN=95 TESTVAL=0.

Reliability

Scale: ALL VARIABLES

Case Processing Summary	
	N	%	
Cases	Valid	31	100.0	
	Excludeda	0	.0	
	Total	31	100.0	

a. Listwise deletion based on all variables in the procedure.	


Reliability Statistics	
Cronbach's Alpha	N of Items	
.999	2	


Intraclass Correlation Coefficient	
	Intraclass Correlationb	95% Confidence Interval	F Test with True Value 0			
		Lower Bound	Upper Bound	Value	df1			
Single Measures	.998a	.995	.999	853.572	30			
Average Measures	.999	.998	.999	853.572	30			

Consistency between observers
RELIABILITY
  /VARIABLES=meanvalueofobserver1 meanvalueofobserver2
  /SCALE('ALL VARIABLES') ALL
  /MODEL=ALPHA
  /ICC=MODEL(RANDOM) TYPE(CONSISTENCY) CIN=95 TESTVAL=0.

Reliability

Scale: ALL VARIABLES

Case Processing Summary	
	N	%	
Cases	Valid	31	100.0	
	Excludeda	0	.0	
	Total	31	100.0	

a. Listwise deletion based on all variables in the procedure.	

Reliability Statistics	
Cronbach's Alpha	N of Items	
.989	2	


Intraclass Correlation Coefficient	
	Intraclass Correlationb	95% Confidence Interval	F Test with True Value 0			
		Lower Bound	Upper Bound	Value	df1			
Single Measures	.979a	.957	.990	93.930	30			
Average Measures	.989	.978	.995	93.930	30			

4. HCOR of preoperative surgical side
Consistency of observer 1
RELIABILITY
  /VARIABLES=observer1first observer1second
  /SCALE('ALL VARIABLES') ALL
  /MODEL=ALPHA
  /ICC=MODEL(RANDOM) TYPE(CONSISTENCY) CIN=95 TESTVAL=0.

Reliability

Scale: ALL VARIABLES

Case Processing Summary	
	N	%	
Cases	Valid	31	100.0	
	Excludeda	0	.0	
	Total	31	100.0	

a. Listwise deletion based on all variables in the procedure.	

Reliability Statistics	
Cronbach's Alpha	N of Items	
.999	2	


Intraclass Correlation Coefficient	
	Intraclass Correlationb	95% Confidence Interval	F Test with True Value 0			
		Lower Bound	Upper Bound	Value	df1			
Single Measures	.998a	.996	.999	1158.033	30			
Average Measures	.999	.998	1.000	1158.033	30			

Consistency of observer 2
RELIABILITY
  /VARIABLES=observer2first observer2second
  /SCALE('ALL VARIABLES') ALL
  /MODEL=ALPHA
  /ICC=MODEL(RANDOM) TYPE(CONSISTENCY) CIN=95 TESTVAL=0.

Reliability
Scale: ALL VARIABLES
Case Processing Summary	
	N	%	
Cases	Valid	31	100.0	
	Excludeda	0	.0	
	Total	31	100.0	

a. Listwise deletion based on all variables in the procedure.	

Reliability Statistics	
Cronbach's Alpha	N of Items	
.998	2	


Intraclass Correlation Coefficient	
	Intraclass Correlationb	95% Confidence Interval	F Test with True Value 0			
		Lower Bound	Upper Bound	Value	df1			
Single Measures	.996a	.993	.998	553.853	30			
Average Measures	.998	.996	.999	553.853	30			

Consistency between observers
RELIABILITY
  /VARIABLES=meanvalueofobserver1 meanvalueofobserver2
  /SCALE('ALL VARIABLES') ALL
  /MODEL=ALPHA
  /ICC=MODEL(RANDOM) TYPE(CONSISTENCY) CIN=95 TESTVAL=0.
Reliability

Scale: ALL VARIABLES


Case Processing Summary	
	N	%	
Cases	Valid	31	100.0	
	Excludeda	0	.0	
	Total	31	100.0	

a. Listwise deletion based on all variables in the procedure.	


Reliability Statistics	
Cronbach's Alpha	N of Items	
.980	2	


Intraclass Correlation Coefficient	
	Intraclass Correlationb	95% Confidence Interval	F Test with True Value 0			
		Lower Bound	Upper Bound	Value	df1			
Single Measures	.960a	.920	.981	49.454	30			
Average Measures	.980	.958	.990	49.454	30			

5. VCOR of postoperative surgical side
Consistency of observer 1
RELIABILITY
  /VARIABLES=observer1first observer1second
  /SCALE('ALL VARIABLES') ALL
  /MODEL=ALPHA
  /ICC=MODEL(RANDOM) TYPE(CONSISTENCY) CIN=95 TESTVAL=0.
Reliability

Scale: ALL VARIABLES

Case Processing Summary	
	N	%	
Cases	Valid	31	100.0	
	Excludeda	0	.0	
	Total	31	100.0	

a. Listwise deletion based on all variables in the procedure.	

Reliability Statistics	
Cronbach's Alpha	N of Items	
.999	2	


Intraclass Correlation Coefficient	
	Intraclass Correlationb	95% Confidence Interval	F Test with True Value 0			
		Lower Bound	Upper Bound	Value	df1			
Single Measures	.997a	.994	.999	669.497	30			
Average Measures	.999	.997	.999	669.497	30			

Consistency of observer 2
RELIABILITY
  /VARIABLES=observer2first observer2second
  /SCALE('ALL VARIABLES') ALL
  /MODEL=ALPHA
  /ICC=MODEL(RANDOM) TYPE(CONSISTENCY) CIN=95 TESTVAL=0.

Reliability

Scale: ALL VARIABLES


Case Processing Summary	
	N	%	
Cases	Valid	31	100.0	
	Excludeda	0	.0	
	Total	31	100.0	

a. Listwise deletion based on all variables in the procedure.	


Reliability Statistics	
Cronbach's Alpha	N of Items	
.998	2	


Intraclass Correlation Coefficient	
	Intraclass Correlationb	95% Confidence Interval	F Test with True Value 0			
		Lower Bound	Upper Bound	Value	df1			
Single Measures	.995a	.990	.998	404.023	30			
Average Measures	.998	.995	.999	404.023	30			

Consistency between observers
RELIABILITY
  /VARIABLES=meanvalueofobserver1 meanvalueofobserver2
  /SCALE('ALL VARIABLES') ALL
  /MODEL=ALPHA
  /ICC=MODEL(RANDOM) TYPE(CONSISTENCY) CIN=95 TESTVAL=0.
Reliability

Scale: ALL VARIABLES
Case Processing Summary	
	N	%	
Cases	Valid	31	100.0	
	Excludeda	0	.0	
	Total	31	100.0	

a. Listwise deletion based on all variables in the procedure.	


Reliability Statistics	
Cronbach's Alpha	N of Items	
.980	2	


Intraclass Correlation Coefficient	
	Intraclass Correlationb	95% Confidence Interval	F Test with True Value 0			
		Lower Bound	Upper Bound	Value	df1			
Single Measures	.960a	.919	.981	49.234	30			
Average Measures	.980	.958	.990	49.234	30			

6. HCOR of postoperative surgical side
Consistency of observer 1
RELIABILITY
  /VARIABLES=observer1first observer1second
  /SCALE('ALL VARIABLES') ALL
  /MODEL=ALPHA
  /ICC=MODEL(RANDOM) TYPE(CONSISTENCY) CIN=95 TESTVAL=0.

Reliability
Scale: ALL VARIABLES

Case Processing Summary	
	N	%	
Cases	Valid	31	100.0	
	Excludeda	0	.0	
	Total	31	100.0	

a. Listwise deletion based on all variables in the procedure.	

Reliability Statistics	
Cronbach's Alpha	N of Items	
.996	2	


Intraclass Correlation Coefficient	
	Intraclass Correlationb	95% Confidence Interval	F Test with True Value 0			
		Lower Bound	Upper Bound	Value	df1			
Single Measures	.993a	.985	.997	277.554	30			
Average Measures	.996	.993	.998	277.554	30			

Consistency of observer 2
RELIABILITY
  /VARIABLES=observer2first observer2second
  /SCALE('ALL VARIABLES') ALL
  /MODEL=ALPHA
  /ICC=MODEL(RANDOM) TYPE(CONSISTENCY) CIN=95 TESTVAL=0.
Reliability

Scale: ALL VARIABLES

Case Processing Summary	
	N	%	
Cases	Valid	31	100.0	
	Excludeda	0	.0	
	Total	31	100.0	

a. Listwise deletion based on all variables in the procedure.	

Reliability Statistics	
Cronbach's Alpha	N of Items	
.999	2	


Intraclass Correlation Coefficient	
	Intraclass Correlationb	95% Confidence Interval	F Test with True Value 0			
		Lower Bound	Upper Bound	Value	df1			
Single Measures	.997a	.994	.999	715.282	30			
Average Measures	.999	.997	.999	715.282	30			

Consistency between observers
RELIABILITY
  /VARIABLES=meanvalueofobserver1 meanvalueofobserver2
  /SCALE('ALL VARIABLES') ALL
  /MODEL=ALPHA
  /ICC=MODEL(RANDOM) TYPE(CONSISTENCY) CIN=95 TESTVAL=0.

Reliability


Scale: ALL VARIABLES

Case Processing Summary	
	N	%	
Cases	Valid	31	100.0	
	Excludeda	0	.0	
	Total	31	100.0	

a. Listwise deletion based on all variables in the procedure.	


Reliability Statistics	
Cronbach's Alpha	N of Items	
.976	2	


Intraclass Correlation Coefficient	
	Intraclass Correlationb	95% Confidence Interval	F Test with True Value 0			
		Lower Bound	Upper Bound	Value	df1			
Single Measures	.953a	.906	.977	41.960	30			
Average Measures	.976	.951	.989	41.960	30			

7.VCOR of contralateral side
Consistency of observer 1
RELIABILITY
  /VARIABLES=observer1first observer1second
  /SCALE('ALL VARIABLES') ALL
  /MODEL=ALPHA
  /ICC=MODEL(RANDOM) TYPE(CONSISTENCY) CIN=95 TESTVAL=0.
Reliability

Scale: ALL VARIABLES

Case Processing Summary	
	N	%	
Cases	Valid	29	100.0	
	Excludeda	0	.0	
	Total	29	100.0	

a. Listwise deletion based on all variables in the procedure.	

Reliability Statistics	
Cronbach's Alpha	N of Items	
.994	2	


Intraclass Correlation Coefficient	
	Intraclass Correlationb	95% Confidence Interval	F Test with True Value 0			
		Lower Bound	Upper Bound	Value	df1			
Single Measures	.988a	.975	.994	168.067	28			
Average Measures	.994	.987	.997	168.067	28			

Consistency of observer 2
RELIABILITY
  /VARIABLES=observer2first observer2second
  /SCALE('ALL VARIABLES') ALL
  /MODEL=ALPHA
  /ICC=MODEL(RANDOM) TYPE(CONSISTENCY) CIN=95 TESTVAL=0.
Reliability

Scale: ALL VARIABLES

Case Processing Summary	
	N	%	
Cases	Valid	29	100.0	
	Excludeda	0	.0	
	Total	29	100.0	

a. Listwise deletion based on all variables in the procedure.	


Reliability Statistics	
Cronbach's Alpha	N of Items	
.996	2	


Intraclass Correlation Coefficient	
	Intraclass Correlationb	95% Confidence Interval	F Test with True Value 0			
		Lower Bound	Upper Bound	Value	df1			
Single Measures	.992a	.983	.996	242.679	28			
Average Measures	.996	.991	.998	242.679	28			

Consistency between observers
RELIABILITY
  /VARIABLES=meanvalueofobserver1 meanvalueofobserver2
  /SCALE('ALL VARIABLES') ALL
  /MODEL=ALPHA
  /ICC=MODEL(RANDOM) TYPE(CONSISTENCY) CIN=95 TESTVAL=0.

Reliability
Scale: ALL VARIABLES
Case Processing Summary	
	N	%	
Cases	Valid	29	100.0	
	Excludeda	0	.0	
	Total	29	100.0	

a. Listwise deletion based on all variables in the procedure.	

Reliability Statistics	
Cronbach's Alpha	N of Items	
.931	2	


Intraclass Correlation Coefficient	
	Intraclass Correlationb	95% Confidence Interval	F Test with True Value 0			
		Lower Bound	Upper Bound	Value	df1			
Single Measures	.870a	.742	.937	14.404	28			
Average Measures	.931	.852	.967	14.404	28			

8. HCOR of contralateral side
Consistency of observer 1
RELIABILITY
  /VARIABLES=observer1first observer1second
  /SCALE('ALL VARIABLES') ALL
  /MODEL=ALPHA
  /ICC=MODEL(RANDOM) TYPE(CONSISTENCY) CIN=95 TESTVAL=0.
Reliability

Scale: ALL VARIABLES
Case Processing Summary	
	N	%	
Cases	Valid	29	100.0	
	Excludeda	0	.0	
	Total	29	100.0	

a. Listwise deletion based on all variables in the procedure.	

Reliability Statistics	
Cronbach's Alpha	N of Items	
.988	2	

Intraclass Correlation Coefficient	
	Intraclass Correlationb	95% Confidence Interval	F Test with True Value 0			
		Lower Bound	Upper Bound	Value	df1			
Single Measures	.977a	.951	.989	84.132	28			
Average Measures	.988	.975	.994	84.132	28			

Consistency of observer 2
RELIABILITY
  /VARIABLES=observer2first observer2second
  /SCALE('ALL VARIABLES') ALL
  /MODEL=ALPHA
  /ICC=MODEL(RANDOM) TYPE(CONSISTENCY) CIN=95 TESTVAL=0.

Reliability

Scale: ALL VARIABLES


Case Processing Summary	
	N	%	
Cases	Valid	29	100.0	
	Excludeda	0	.0	
	Total	29	100.0	

a. Listwise deletion based on all variables in the procedure.	


Reliability Statistics	
Cronbach's Alpha	N of Items	
.988	2	


Intraclass Correlation Coefficient	
	Intraclass Correlationb	95% Confidence Interval	F Test with True Value 0			
		Lower Bound	Upper Bound	Value	df1			
Single Measures	.976a	.950	.989	82.261	28			
Average Measures	.988	.974	.994	82.261	28			

Consistency between observers
RELIABILITY
  /VARIABLES=meanvalueofobserver1 meanvalueofobserver2
  /SCALE('ALL VARIABLES') ALL
  /MODEL=ALPHA
  /ICC=MODEL(RANDOM) TYPE(CONSISTENCY) CIN=95 TESTVAL=0.

Reliability

Scale: ALL VARIABLES

Case Processing Summary	
	N	%	
Cases	Valid	29	100.0	
	Excludeda	0	.0	
	Total	29	100.0	

a. Listwise deletion based on all variables in the procedure.	

Reliability Statistics	
Cronbach's Alpha	N of Items	
.817	2	


Intraclass Correlation Coefficient	
	Intraclass Correlationb	95% Confidence Interval	F Test with True Value 0			
		Lower Bound	Upper Bound	Value	df1			
Single Measures	.690a	.439	.842	5.461	28			
Average Measures	.817	.610	.914	5.461	28			
